# Understanding Venous Remodeling After Minimally Invasive and Noninvasive Treatments for Varicose Veins: A Mechanistic Narrative Review

**DOI:** 10.3390/medsci14060691

**Published:** 2026-10-21

**Authors:** Nicolas Falvo, François Ghiringhelli, Olivier Chevallier, Chiara Longo, Valentin Derangère, Romaric Loffroy

**Affiliations:** 1 Vascular Medicine Unit, Department of Diagnostic and Interventional Imaging, Image-Guided Therapy Center, CHU François-Mitterrand, 21079 Dijon Cedex, France; 2 Centre Georges-François Leclerc (CGFL), Translational Platform in Cancer Biology, INSERM U1231, Therapies and Immune Response in Cancers (TIRECS), 21000 Dijon, France; 3 Department of Diagnostic and Interventional Imaging, Image-Guided Therapy Center, CHU François-Mitterrand, 21079 Dijon Cedex, France; 4 ICMUB Laboratory, UMR CNRS 6302, CHU François-Mitterrand, 21079 Dijon Cedex, France

**Keywords:** venous ablation, venous remodeling, histopathology, immunohistochemistry, fibrosis, recanalization

## Abstract

The absence of detectable flow on duplex ultrasound after venous ablation may correspond to different tissue states. This mechanistic narrative review examines histopathological and immunohistochemical responses after thermal, chemical, mechanochemical, adhesive, and focused ultrasound treatments. The PubMed search, conducted on 6 September 2026 for publications available through 31 August 2026, resulted in the inclusion of 45 tissue-based publications; six additional references provided clinical context. Study selection and data extraction were performed by a single author. Human and experimental data were organized by wall compartment, treatment modality, and time point. Depending on the technique, the initial signatures comprised thermal injury, endothelial detergent toxicity, mechanical abrasion, adhesive polymerization, or cavitation. Luminal organization, fibrosis, and recanalization followed heterogeneous trajectories. Cyanoacrylate was distinguished by a biomaterial response with variable persistence. Early occlusion does not demonstrate mature fibrous obliteration. The depth–continuity–topography framework is a working hypothesis without validated predictive value. Combining longitudinal duplex ultrasound follow-up with end-point histological analysis of the same segment, including morphometry, immunohistochemistry, and matrix assessment, could better relate tissue status to clinical outcome.

## 1. Introduction

Since the clinical introduction of endovenous laser therapy in the early 2000s [1], endovenous treatments have profoundly changed the management of superficial venous reflux. International guidelines favor minimally invasive techniques when indicated [2]. Their efficacy and safety are assessed primarily using clinical and ultrasound criteria: elimination of reflux, absence of flow in the treated truncal vein, reduction in diameter, improvement in symptoms, and adverse events. These criteria are essential for patient follow-up but do not directly characterize the biological state of the treated vein.

A noncompressible vein with no detectable flow may contain a recent coagulum, an organizing thrombus, vascularized granulation tissue, a collagenous scar, or a polymer. Microscopic channels may also be present without generating detectable flow on duplex ultrasound. The term “venous closure” therefore encompasses several tissue states with differing stability and reversibility. Distinct technologies may produce the same ultrasound endpoint without inducing the same wall remodeling (Figure 1).

Endovenous laser ablation (EVLA) and radiofrequency ablation (RFA) induce thermal injuries that depend on the device, the energy delivered, and its transfer to the wall [3,4,5,6,7,8,9,10,11]. Sclerosants initially target the endothelium; their effect on the media varies according to the agent, concentration, liquid or foam formulation, and contact conditions [12,13,14,15]. Mechanochemical ablation (MOCA) adds a mechanical action that facilitates wall exposure to the sclerosant [16,17,18]. Cyanoacrylate achieves closure through polymerization and wall apposition, followed by a biomaterial response [19,20,21]. Thermal and mechanical high-intensity focused ultrasound (HIFU) complete this spectrum through a transcutaneous approach without an endoluminal device [22,23,24,25].

The available histological evidence is scattered across animal models, ex vivo human segments, opportunistic specimens, and experimental studies using heterogeneous devices or energy settings. Assessment time points range from a few minutes to several years. Outcomes are often qualitative, and the individual wall layers and luminal contents are not always examined separately. A synthesis centered on vein-wall biology may therefore usefully complement conventional clinical comparisons.

This review aims to describe changes in the lumen and wall after the main venous ablation modalities, analyze their evolution over time, and propose a common mechanistic framework. This framework distinguishes early occlusion, mature fibrous obliteration, and endothelialized recanalization. The guiding question is as follows: can the same ultrasound-defined occlusion correspond to markedly different biological states? Tissue observations reported in the literature are distinguished from the proposed explanatory framework, whose predictive value remains to be established.

## 2. Methods

### 2.1. Review Design and Question

This mechanistic narrative review, based on a structured literature search, examines the mechanisms of venous remodeling across the different wall compartments. This approach reflects the heterogeneity of the available evidence: in vivo or ex vivo human studies, animal models, in vitro experiments, devices and technical parameters that vary across studies, opportunistic specimens, and variable histological time points. The objective was not to compare the overall clinical efficacy of the techniques, but to describe the nature, depth, and continuity of the lesions and their evolution over time.

The central question was as follows: after thermal, chemical, mechanical, adhesive, or acoustic venous ablation, what changes occur in the lumen, endothelium, intima, media, adventitia, and extracellular matrix? We also searched for trajectories that could lead either to durable fibrous obliteration or to endothelial restoration and recanalization.

### 2.2. Information Sources and Search Strategy

A literature search was conducted in PubMed on 6 September 2026 for publications available through 31 August 2026, with no restrictions on language, species, or study type. The query combined terms related to superficial veins and venous insufficiency, ablation modalities, and tissue responses. The main keywords, complete search string, and results are provided in Supplementary Material S1.

PubMed was selected for its coverage of biomedical literature on venous treatments and tissue responses. Its MeSH indexing, combined with title and abstract terms, allowed the search strategy to be documented in detail.

### 2.3. Eligibility Criteria

We included studies directly describing the tissue response of a vein after treatment by conventional histology, matrix staining, electron microscopy, immunohistochemistry, immunofluorescence, molecular analyses, or digital morphometry. Human, animal, ex vivo, and in vitro studies were eligible provided that the ablation technique and the vein studied were clearly identified.

- The techniques studied included EVLA, RFA, liquid or foam sclerotherapy, MOCA, cyanoacrylate, steam, and thermal or mechanical focused ultrasound.
- Outcomes had to be attributable to the luminal contents, endothelium, intima, media, adventitia, perivenous tissues, or extracellular matrix.
- The device, agent, dose, or energy parameters had to be described in sufficient detail to allow mechanistic interpretation.
- Full original articles were prioritized. Case series and late histological observations were retained when they provided information not available from other models.

Exclusively clinical studies without tissue or molecular data were excluded from the histopathological synthesis, as were unsupported opinion articles and studies in which the vein or technique could not be identified. Guidelines and selected clinical studies were nevertheless cited to provide context for closure, reflux, and recurrence; they were not used as histological evidence. Secondary reviews were used to identify additional primary studies.

Studies meeting the eligibility criteria were included, even when their findings agreed with those of other reports. They could thus provide independent replication or complement existing observations through a different model, protocol, or time point. When a secondary review contained original tissue observations, only those observations were extracted; studies cited within that review were not counted again.

### 2.4. Study Selection, Data Extraction, and Organization

Nicolas Falvo performed the literature search, primary study selection, and data extraction. Articles with uncertain eligibility were reread before the final decision. François Ghiringhelli and Romaric Loffroy contributed to review conception, discussion of study selection, and synthesis. Primary selection and extraction therefore relied on a single author.

Data extraction covered the model, species, documented sample sizes, treatment parameters, assessment time point, stains, and markers. It also recorded findings concerning the lumen, wall, inflammation, matrix, and any biomaterials. The numbers of subjects, veins, sections, and images were recorded separately to distinguish the units of analysis. The tissue-based studies are summarized in the study tables below.

For readability, observations were grouped into five indicative periods: immediate (up to 24 h), early (>24 h to 7 days), intermediate (>7 days to 6 weeks), remodeling (>6 weeks to 6 months), and late (>6 months). These boundaries structure the synthesis without defining universal biological stages; exact time points are reported when available. Late human specimens, which were often obtained opportunistically, document the existence of a tissue state without estimating its frequency.

### 2.5. Synthesis

The synthesis was qualitative and mechanistic. It compared the initial injury, lesion depth and continuity, inflammatory response, luminal organization, fibrosis, contraction, and recanalization. Heterogeneity in models, techniques, and time points precluded a meaningful meta-analysis or causal ranking of treatments. Limitations were assessed descriptively, without a formal risk-of-bias score. Findings reported directly by the studies were distinguished from mechanistic interpretations and working hypotheses. The proposed framework distinguishes fibrous obliteration from recanalization while accounting for intermediate states and the distinct trajectory of adhesive treatments.

### 2.6. Studies Included in the Synthesis

The PubMed search identified 698 publications, of which 54 underwent full-text assessment. Of these, 45 met the eligibility criteria. The two study tables present 20 and 26 publications, respectively. One study with both a laser arm and a foam arm appears in both tables and is counted only once in the total. Six clinical-context or guideline references complement the synthesis, bringing the bibliography to 51 references.

## 3. Vein-Wall Compartments and Response to Injury

### 3.1. Endothelium and Intima

The endothelium forms the interface between the blood and the vessel wall. It contributes to the regulation of hemostasis, vascular tone, leukocyte adhesion, and permeability. Thermal, chemical, and mechanochemical techniques generally alter the endothelium during the initial phase [3,4,5,6,7,8,12,13,14,15]. Cyanoacrylate differs because initial closure results from wall apposition and adhesive polymerization [19,20,21]. In all cases, isolated endothelial denudation is insufficient to ensure durable closure. Viable endothelial islands, discontinuous injury, or regrowth from preserved segments may promote recanalization.

The intima of varicose veins is already heterogeneous: it may be thickened, disorganized, or exhibit matrix alterations. Comparisons with healthy veins must therefore be interpreted cautiously. Endothelial markers such as von Willebrand factor (vWF), CD31, or ERG help identify endothelial cells. It is then necessary to determine whether the identified endothelial cells line a true channel and to examine its shape and continuity across multiple sections. A small reparative vessel within organized tissue does not, by itself, demonstrate that blood can again flow through the treated truncal vein.

The experiments by Bottaro et al. on endothelial cells and ex vivo human umbilical veins illustrate this distinction: assessment of polidocanol foams combined measurements of loss of cell adhesion and endothelial permeability [26]. These acute endpoints document the effect on the blood–wall interface but provide no information on the continuity of a healed lesion or late fibrous obliteration.

### 3.2. Media, Smooth Muscle Cells, and Matrix

The media consists mainly of smooth muscle cells, collagen, and elastic fibers. After thermal treatment, protein denaturation and cell death may cause immediate wall contraction followed by fibrous reorganization. After sclerotherapy, medial injury is often less immediate. It depends in particular on the agent concentration, wall contact time, and secondary inflammatory or apoptotic mechanisms [12,13,14,15].

Alpha-smooth muscle actin (α-SMA) provides information on the phenotype of smooth muscle cells and myofibroblasts. Its expression depends on the compartment and time point and, in isolation, is not a viability test. Masson trichrome, Sirius red, and elastic fiber stains describe matrix composition and organization. Matrix metalloproteinases MMP-2 and MMP-9 and their tissue inhibitors (TIMPs) could complement this assessment, but their expression does not directly measure enzymatic activity. Their value for predicting the outcome of a treated vein remains to be established.

### 3.3. Adventitia, Inflammation, and Perivenous Tissues

The adventitia is not merely a supporting envelope; it is an active immune and matrix compartment. Extension of the lesion into this layer may contribute to healing, but excessive depth also exposes the perivenous tissues. The inflammatory response varies by technique: sterile inflammation after thermal treatment, macrophage recruitment during thrombus organization, a foreign-body giant-cell granulomatous reaction around cyanoacrylate, or inflammation associated with cavitation and mechanical disruption [5,10,15,19,20,21,25].

The presence of CD68+ or CD163+ macrophages, giant cells, lymphocytes, or neutrophils cannot be considered favorable or unfavorable in isolation. Interpretation depends on the time after treatment, cell location, possible persistence of a material, and the quality of fibrous organization. Treatment selectivity must therefore be assessed in both the targeted wall and the surrounding tissues.

### 3.4. From Luminal Contents to Fibrous Organization

When treatment disrupts the endothelium, matrix exposure promotes platelet activation and coagulation. Early luminal contents may therefore contribute to the absence of flow, but their persistence does not indicate integration into the wall. Organization involves cellular infiltration, transient neovascularization, progressive collagen deposition, and fusion between the luminal contents and the wall. Cyanoacrylate initially follows a different pathway but likewise leads to chronic inflammation and fibrous organization around the material. Depending on lesion continuity, biomaterial persistence, and endothelial survival, the outcome may be a compact scar or reopening of a passage through the treated segment that allows blood to flow again (recanalization) [5,9,16,19,20,21].

## 4. Modality-Specific Tissue Signatures

### 4.1. Endovenous Laser Ablation

EVLA converts light energy into heat. Energy is absorbed by components of the blood and wall known as chromophores, whose absorption varies with the laser wavelength. Intraluminal blood, the formation of steam or carbonized areas, fiber-to-wall contact, fiber centering, and emission geometry all influence energy distribution [3,4,5,6,7,8,9]. Ex vivo and animal models show endothelial denudation, protein coagulation, disruption or vacuolization of the media, and occasionally focal adventitial injury. The lesion may therefore be deep at the point of contact and more limited on the opposite wall.

Fiber centering and radial emission geometries are intended to improve energy distribution. However, linear energy density alone does not describe the energy actually received by the wall. Residual blood, pullback speed, and fiber-to-wall contact influence lesion distribution [3,4,5,6,7,8]. A transverse section provides information on circumferential continuity; only analysis at multiple levels can approximate longitudinal continuity. Preserved areas may contribute to recanalization.

In ex vivo human saphenous segments, comparison of a 1470 nm laser with a two-ring radial fiber and RFA showed different morphological patterns of thermal denaturation according to the device and laser energy [27]. Azan trichrome staining allows lesion depth to be measured but demonstrates neither loss of viability across the full wall thickness nor the late outcome of the segment.

Over time, the wall and luminal contents may evolve into connective tissue, with a variable reduction in diameter. In an ovine model using a 1910 nm laser, connective tissue transformation increased with dose, but occlusion remained incomplete [9]. Histological change should therefore not be equated with durable closure. The use of healthy animal veins without reflux limits extrapolation to human varicose saphenous veins.

Two ex vivo human studies describe lesions produced by a 1470 nm radial laser in a vein from a single donor and by a 980 nm laser in eleven veins analyzed as paired halves [28,29]. In the latter, CCND1, BCL2, and BAX analysis adds a molecular dimension whose relationship with functional viability remains to be established [29]. A clinical study comparing, among other treatments, laser and foam also describes wall changes, but the sampling time is insufficiently documented to place them within a healing trajectory [30].

### 4.2. Endovenous Radiofrequency Ablation

RFA delivers controlled thermal energy. Devices differ: segmental systems transfer heat generated by a heating element to the wall, whereas electrode-based devices rely on tissue resistive heating. Collagen denaturation is accompanied by contraction, followed by cellular injury and fibrous organization. Under the experimental conditions studied, some reports describe more homogeneous injury than with earlier laser fibers [3,10,11]. This observation cannot be generalized to all device generations.

A controlled temperature does not ensure identical lesion depth along the entire vein. The wall is heterogeneous, the catheter may be eccentric, and junctions alter local geometry. Histological analysis should therefore describe not only the mean depth of injury but also its continuity around the circumference and along the treated segment.

A canine study comparing ClosureFAST (Medtronic, Minneapolis, MN, USA) and VENISTAR (STARmed Co., Ltd., Goyang-si, Republic of Korea; thermal ablation using bipolar radiofrequency) analyzed 69 sites across 23 veins in seven dogs [31]. Immediate specimens were assessed without tumescence, whereas day-14 specimens were assessed with tumescence. This protocol difference limits interpretation of changes over time.

### 4.3. Liquid and Foam Sclerotherapy

Detergents such as polidocanol and sodium tetradecyl sulfate (STS) disrupt cell membranes and initially target the endothelium. Studies of human veins in vivo, ex vivo, or in vitro describe rapid endothelial loss, intimal separation, and, depending on the model, early thrombus formation [12,13,14]. Lesion extent depends in particular on the agent, its concentration, the sclerosant-to-blood ratio, contact time, and emptying of the vein.

Medial injury does not necessarily result from immediate chemical diffusion. After STS exposure, immunofluorescence findings suggest a delayed inflammatory and apoptotic component, particularly after 24 h in culture [15]. This response should not be extrapolated indiscriminately to polidocanol. Acute analysis may underestimate some secondary lesions; conversely, partial loss of staining alone cannot quantify cell death or predict recanalization.

Foam increases the contact surface with the endothelium and displaces part of the blood within the vein. Its distribution nevertheless remains influenced by vein diameter, tributaries, patient position, and bubble characteristics. The presence of thrombus after injection is therefore insufficient to define mature closure, which requires fibrous organization integrated into the wall.

An experimental formulation combining a tranexamic acid-conjugated surfactant and a foam stabilizer (AEO15-TA/PVA) has also been studied in cellular models and mouse and rabbit veins [32]. Histology, immunofluorescence, and matrix analyses showed vascular injury followed by short-term collagen deposition. These preclinical data extend the assessment of sclerotherapy to remodeling but do not demonstrate durable obliteration in humans.

In six ex vivo human GSVs, analysis of paired segments after five minutes of contact with polidocanol foam, with or without glycerine, describes acute effects on the endothelium and media [33]. Rat and rabbit models using different sclerosants also examine venous injury and surrounding tissues [34,35,36]. Their sample sizes and time points remain difficult to compare, particularly when reporting is inconsistent. In humans, an illustration after treatment using the Triplex protocol (three-step chemical sclerotherapy) shows remodeling without occlusion at 30 days [37]. Because the total histological sample size and stains are insufficiently documented, this observation mainly provides an example of a tissue response.

### 4.4. Mechanochemical Endovenous Ablation

The studies presented mainly address MOCA using ClariVein^®^ (Merit Medical Systems, South Jordan, UT, USA), which combines a rotating wire with sclerosant injection. The mechanical action is intended to disrupt the endothelium, induce spasm, and facilitate wall exposure to the sclerosant. In a caprine model, ClariVein^®^ combined with 1.5% STS resulted in fibrous occlusion at 12 weeks [16]. Ex vivo human models also showed deeper endothelial and medial injury than with sclerosant alone, consistent with facilitated penetration of the agent [17,18]. These observations do not necessarily apply to all mechanochemical devices.

A human observation at 1 year complements these findings: the harvested saphenous vein was obliterated, with circumferential loss of the endothelial lining, fibrosis, and medial injury. Microvessels were present within the organized tissue without forming a functional venous lumen [38]. This case documents late remodeling after MOCA but does not establish its frequency or the superiority of this modality.

An ovine model using Flebogrif^®^ (Balton, Warsaw, Poland), a retractable-hook device combined with 3% polidocanol foam, provided a different observation: at 1 month, treated veins showed a restored endothelial lining and newly formed collagen within the wall, with little change in the adventitia [39]. This coexistence illustrates remodeling distinct from complete endothelial loss; by itself, it demonstrates neither functional recanalization nor durable obliteration.

The mechanical effect nevertheless depends on contact between the wire and the wall and therefore on vein diameter, catheter position, and the degree of spasm achieved. The sclerosant dose also remains a key determinant. MOCA should therefore be understood as an interaction between abrasion, reduction in blood volume, and increased wall exposure to the agent.

### 4.5. Cyanoacrylate Endovenous Closure

Cyanoacrylate differs from the other modalities. Initial closure is based primarily on wall apposition and adhesive polymerization, without requiring heat or extensive thrombosis. The persistent material subsequently triggers a chronic inflammatory response involving macrophages, multinucleated giant cells, encapsulation, and fibrosis [19,20,21].

Late specimens show heterogeneous evolution. After VenaSeal™ (Medtronic, Minneapolis, MN, USA), a specimen obtained at 5.5 years showed a collagenous scar with polymer remnants, giant cells, and granulomatous foci [20]. At 2 years, another specimen showed a largely preserved media, giant cells, and no adventitial infiltration [21]. In the series using Histoacryl^®^ (B. Braun Melsungen AG, Melsungen, Germany) with Lipiodol^®^ (Guerbet, Villepinte, France), the material was no longer identifiable in the specimen obtained at 3 years [19]. Time point, formulation, and sampling context must therefore be considered separately.

Inflammatory topography is also illustrated by a vein removed on day 200 because of persistent hypersensitivity after cyanoacrylate closure: the predominantly CD4+ lymphocytic infiltrate was mainly luminal, with relative preservation of the peripheral wall [40]. This observation documents an immune profile in the setting of a complication but cannot estimate its frequency after treatment.

Adhesive closure therefore requires neither complete destruction of the media nor disappearance of the biomaterial by a given time point. The persistence of an encapsulated polymer may contribute to tissue evolution without being a universal requirement. The proposed maturity criteria for nonadhesive treatments must be adapted to this modality.

Human specimens obtained for complications also illustrate the diversity of cyanoacrylate responses: wall and perivenous reaction at day 77, puncture-site granulomas, aseptic abscess, and an extravascular mass at nine months [41,42,43,44,45]. Sampling time should be distinguished from symptom-onset time. These observations describe local responses in selected settings, whose frequency and representativeness among treated veins remain unknown.

### 4.6. Thermal and Mechanical Focused Ultrasound

HIFU delivers transcutaneous energy focused on the vein. Thermal systems aim to heat the wall to induce coagulation and contraction. The in vitro human study by Pichardo et al. mainly documented wall retraction without demonstrating durable closure [22]. Ovine experiments provided observations of occlusion and subsequent remodeling [23]. Accurate focal targeting and protection of the skin, nerves, and perivenous tissues remain essential.

Nonthermal mechanical approaches use cavitation to injure the vein. A preclinical study showed that in vivo occlusion could be achieved without microbubble injection [24]. An ovine histological study published in 2026 described consistent destruction of the intima, medial fibrosis, and protocol-dependent adventitial injury at 10 days. Perivenous effects were limited but not absent [25]. These findings remain preliminary because of the small sample sizes and short follow-up.

Focused ultrasound extends venous ablation beyond devices introduced into the lumen. Most importantly, it distinguishes two mechanisms—thermal coagulation and mechanical cavitation—while placing spatial precision at the center of treatment efficacy and safety.

A protocol combining pulsed HIFU, microbubbles, and fibrinogen was studied in seven rabbits [46]. All seven segments were occluded acutely. At day 14, the six animals assessed had four nonocclusive thrombi and two segments without thrombus. Acute closure was therefore not accompanied by demonstrated mature fibrous obliteration. The combined use of the three components also limits attribution of these findings to ultrasound alone. The principal tissue signatures are summarized in Figure 2.

## 5. From Initial Injury to Tissue Outcome: Cross-Modality Synthesis

### 5.1. Immediate and Early Events

During the first hours, the techniques differ primarily in the nature of the initial injury: thermal coagulation, detergent action, abrasion combined with a sclerosant, adhesive polymerization, or cavitation. Except with cyanoacrylate, endothelial disruption is common. It may be accompanied by matrix exposure, coagulation activation, loss of medial cells, and mechanical alteration of the wall. However, the relative contributions of thrombus, wall contraction, and wall apposition vary by technique [3,4,5,6,7,8,9,10,11,12,13,14,15,16,17,18,19,20,21,22,23,24,25].

Analysis of this early phase must distinguish the luminal contents from the wall itself. Luminal filling alone does not establish that endothelial injury is continuous, that the media is affected, or that the luminal contents will evolve into a collagenous scar. Conversely, a severely injured wall may still surround transient luminal spaces before their organization.

### 5.2. Intermediate Organization and Late Remodeling

Over the following weeks, the available observations describe variable organization of the luminal contents, cellular infiltration, and matrix transformation. However, the precise sequence of these events has not been documented for every technique. Collagen may progressively occupy the lumen as medial architecture changes and the interface between the luminal contents and wall becomes less distinct. Around cyanoacrylate, this response develops in contact with the material. Reduction in diameter may reflect initial contraction, scar retraction, or partial resorption of the luminal contents [5,9,16,19,20,21,23,25,38].

At later time points, fibrous obliteration and recanalization represent two possible outcomes. The former combines collagenous organization with loss of a functional venous lumen; the latter corresponds to reopening of a luminal passage after initial closure. Persistent patency, without documented initial closure, should be distinguished from recanalization using longitudinal duplex ultrasound follow-up. Mixed states may occur. Microvessels within reparative tissue must be distinguished from a recanalization channel communicating with the circulation. Clinical data document patency or reflux but do not directly demonstrate endothelialization of the observed channel [5,9,19,20,21,38,47,48,49].

### 5.3. Depth, Continuity, and Topography of Injury

A deep lesion may remain discontinuous, and a mean measurement may conceal preserved areas. Contact and centering models show this variability in distribution [3,5,6,7,8,17,18,28,31]. These observations lead us to propose a common approach to interpreting lesions based on their depth, continuity around and along the vein, and topography (Figure 3). Acute morphometric comparisons complement this approach [30,31,33,50], but their relationship with late closure remains to be established. This framework is a working hypothesis; its predictive value and any technique-specific thresholds will require assessment.

Topography adds a third dimension. Adventitial involvement remains an injury to the vein wall; by itself, it does not demonstrate off-target damage. Off-target damage corresponds to extension beyond the vein to nerves, skin, or adjacent tissues. This distinction should be examined for each modality while balancing venous closure against preservation of the surrounding tissues.

### 5.4. Defining Biological Treatment Maturity

“Biological maturity” is used here as a working concept. It could combine loss of a functional venous lumen, matrix organization, integration of the luminal contents into the wall, and stability over time without excessive perivenous damage. Channel morphology and possible communication with the circulation should be interpreted alongside ultrasound follow-up. No universal collagen, depth, or continuity threshold has currently been established within this framework.

In this review, occlusion denotes the absence of detectable flow in the treated segment at the time of examination and does not establish the nature of the luminal contents. Fibrous obliteration describes an organized histological state with loss of a functional venous lumen. Persistent patency denotes a passage that remained open without documented initial closure, whereas recanalization denotes reopening after initial closure. These definitions require ultrasound endpoints, histological observations, and presumed mechanisms to be distinguished.

After cyanoacrylate treatment, an encapsulated polymer may persist or become less identifiable depending on the time point and formulation. After a nonadhesive technique, poorly organized intraluminal contents may suggest incomplete maturation, but this finding has no validated prognostic value. Comparisons among techniques should therefore respect their specific trajectories rather than seek a single histological phenotype (Table 1) [19,20,21].

## 6. Clinical Implications, Limitations, and Perspectives

### 6.1. Relating Imaging Findings to Tissue Status

Duplex ultrasound remains the reference examination for documenting closure of a treated vein and detecting recurrence [2]. However, it provides an indirect representation of tissue evolution. Reduction in diameter, echogenicity, compressibility, and their changes over time may suggest maturation but cannot reliably distinguish an organized thrombus, a collagenous scar, or persistent material.

Relating imaging to tissue findings requires follow-up of closure and reflux in the same segment until sampling. This follow-up distinguishes initial closure, persistent patency, and recanalization. The interval between the last duplex examination and sampling should be specified, and tissue observations considered alongside symptoms and any reinterventions. This comparison could reveal correspondences between remodeling and clinical outcome, with causality still to be established.

Recanalization probably does not result from a single mechanism. It may arise from insufficient or discontinuous initial injury, endothelial restoration, channel formation within a thrombus, or hemodynamic constraints. Its clinical impact is also variable: it may remain partial and minimally symptomatic, but it may also be associated with reflux, clinical recurrence, or reintervention [47,48,49]. The available studies rely mainly on clinical follow-up and duplex ultrasound. They do not demonstrate that a recanalized vein recovers a normal wall and normal valvular function. Combining histological analysis with longitudinal color duplex ultrasound follow-up is therefore necessary to relate tissue status to reflux and clinical durability.

The inflammatory response must be interpreted according to time. Early inflammation may contribute to organization and healing. Conversely, persistent inflammation around a biomaterial or excessive extension into perivenous tissues may become undesirable. The presence of immune cells alone is therefore not a criterion of success or failure [15,19,20,21,25].

Clinical translation must also account for vein- and treatment-specific characteristics: initial wall thickness, diameter, intraluminal blood volume, presence of reflux, tributaries, and device centering. These variables may alter lesion depth and continuity even when the technical parameters appear comparable [5,6,7,8,9,10,11,16,23,24,25,50].

### 6.2. Limitations of the Synthesis and Translation of Experimental Models

The level of evidence remains highly uneven across techniques. Some are supported by several experimental studies, whereas others are documented only by a few late human specimens. The number of publications therefore does not allow conclusions regarding the clinical efficacy or biological superiority of a modality. This review compares reported tissue signatures and trajectories rather than causal effects.

Each model provides different information. Controlled experiments describe lesions under defined conditions; ex vivo segments mainly inform acute effects; opportunistic human specimens document tissue states in their clinical context. Cyanoacrylate complication cases thus show venous, perivenous, or puncture-site responses without allowing their frequency to be measured [41,42,43,44,45]. Combined protocols and experimental agents should likewise be considered under their specific conditions, with translation to human efficacy and safety still to be established [34,35,36,37,46].

Treatment parameters are also highly heterogeneous: wavelength, fiber type, energy, number of cycles, sclerosant concentration, contact time, foam formulation, adhesive volume, vein diameter, and emptying conditions. Two devices within the same category may therefore produce different lesions.

Ex vivo studies allow some contact conditions to be controlled but do not reproduce the full circulation, immune response, and healing process. Some models nevertheless retain perivenous tissues or use experimental reperfusion [3]. Animal models most often use healthy veins without reflux, whose wall and hemodynamics differ from those of human varicose saphenous veins. Late human specimens, often obtained in atypical situations, cannot estimate the frequency of the observed states.

Small sample sizes and the diversity of models and time points limit comparison of the findings. Primary selection and extraction by a single author, without an independent duplicate procedure, constitute another limitation. Because the search was restricted to PubMed, studies indexed in other databases may have been missed. These limitations explain the descriptive nature of the synthesis and the need for prospective validation of the proposed relationships between histological profiles and venous outcomes.

### 6.3. Toward Standardized Tissue Assessment

The priority is to standardize sampling and the unit of analysis. Proximal, middle, and distal specimens identified before treatment would allow circumferential and longitudinal variations to be described. The analysis should distinguish the subject, vein, segment, section, and field and account for this hierarchy in statistical comparisons. Digital morphometry would complement expert assessment with measurements of luminal area, wall thickness, collagen content, and spatial variability.

Each vein could undergo duplex ultrasound follow-up until end-point histological sampling of its identified segment. Tissue changes over time would then be studied by comparing separate groups sampled at different time points. This comparison of ultrasound follow-up and tissue findings would allow the depth–continuity–topography framework to be tested while accounting for diameter, reflux, tributaries, and device parameters.

An experimental model allows this follow-up to be planned, the segment to be identified before treatment, and sampling to be performed at a prespecified time point. In humans, access to tissue generally depends on a clinical indication, such as reintervention, a complication, or another indicated operation. The sampled segment must be identifiable as the one followed by duplex ultrasound. These specimens can complement experimental observations, but their selection cannot establish how often these profiles occur among all treated veins.

A core panel combining conventional histology, collagen staining, an endothelial marker, and α-SMA could be supplemented, depending on the question, by macrophage, elastic fiber, or enzymatic analyses. Antibodies and controls should be adapted to the species and tissue. Marker expression, the presence of a cell, and its biological activity must remain distinct concepts. Proposed thresholds and scores will require validation before any predictive use. The included studies are presented in Table 2.

Publication [30] appears in both tables for its laser and foam arms and is counted only once among the 45 distinct publications. “Not reported” indicates information not provided in usable detail; “acute” indicates a phase without an available exact time point. Combined protocols, complication cases, and experimental agents are interpreted separately.

The proposed assessment panel is summarized in Table 3.

## 7. Conclusions

Treatments for superficial venous reflux produce different tissue responses. Thermal injury from EVLA and RFA, the detergent action of sclerotherapy, its combination with mechanical injury in MOCA, the response to cyanoacrylate, and the thermal or mechanical effects of HIFU may all result in the same absence of detectable flow. However, this ultrasound finding alone does not describe the nature or stability of remodeling.

Comparison of the observations leads us to propose a common approach based on lesion depth, continuity, and topography. It could help relate the initial injury to fibrous organization or recanalization, with predictive value still to be established. Interpretation should remain specific to each modality: after adhesive treatment, closure may coexist with a preserved media and prolonged biomaterial evolution.

Combining duplex ultrasound follow-up of an identified segment with its end-point histological analysis, including morphometry, immunohistochemistry, and matrix assessment, could support translational research. This approach could better define correspondences between imaging, luminal composition, and wall remodeling. It could thus contribute to a biological definition of success that complements clinical criteria and distinguishes early occlusion from durable closure.

## Figures and Tables

**Figure 1:**
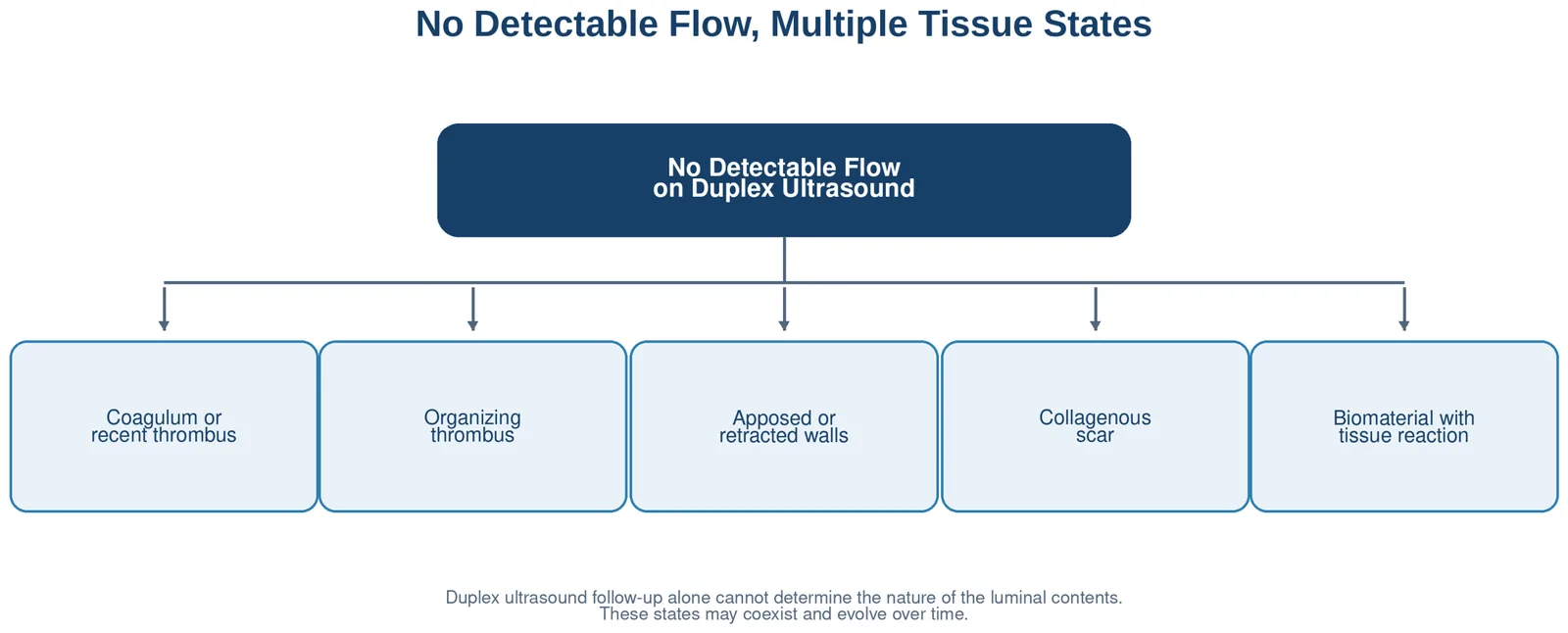
Tissue states that may correspond to an absence of detectable flow on duplex ultrasound. These states may coexist and are not necessarily sequential.

**Figure 2:**
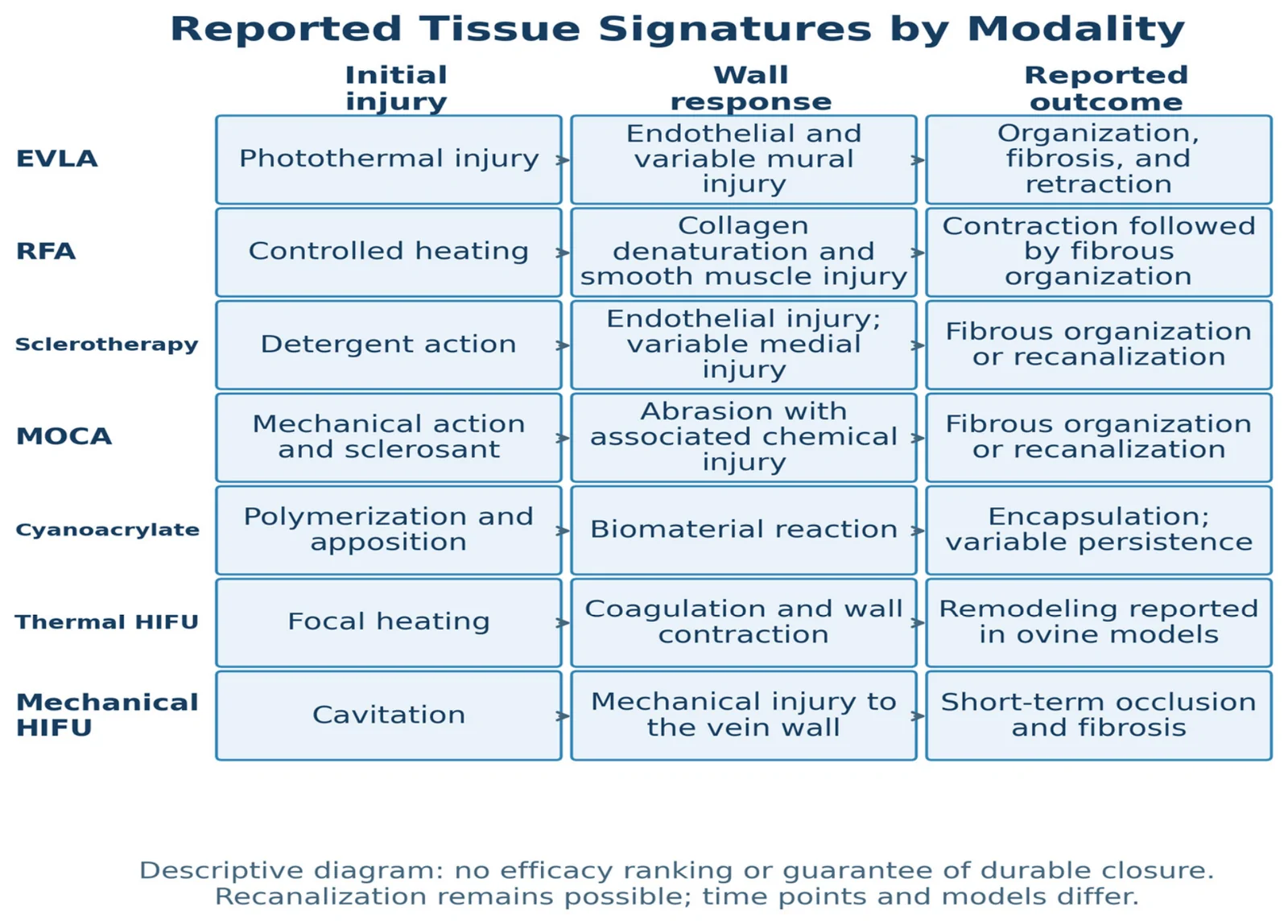
Main tissue signatures reported by modality. The arrows depict mechanistic relationships and do not guarantee a favorable outcome. Recanalization remains possible; the data and follow-up durations are heterogeneous. EVLA: endovenous laser ablation; RFA: radiofrequency ablation; MOCA: mechanochemical ablation; HIFU: high-intensity focused ultrasound.

**Figure 3:**
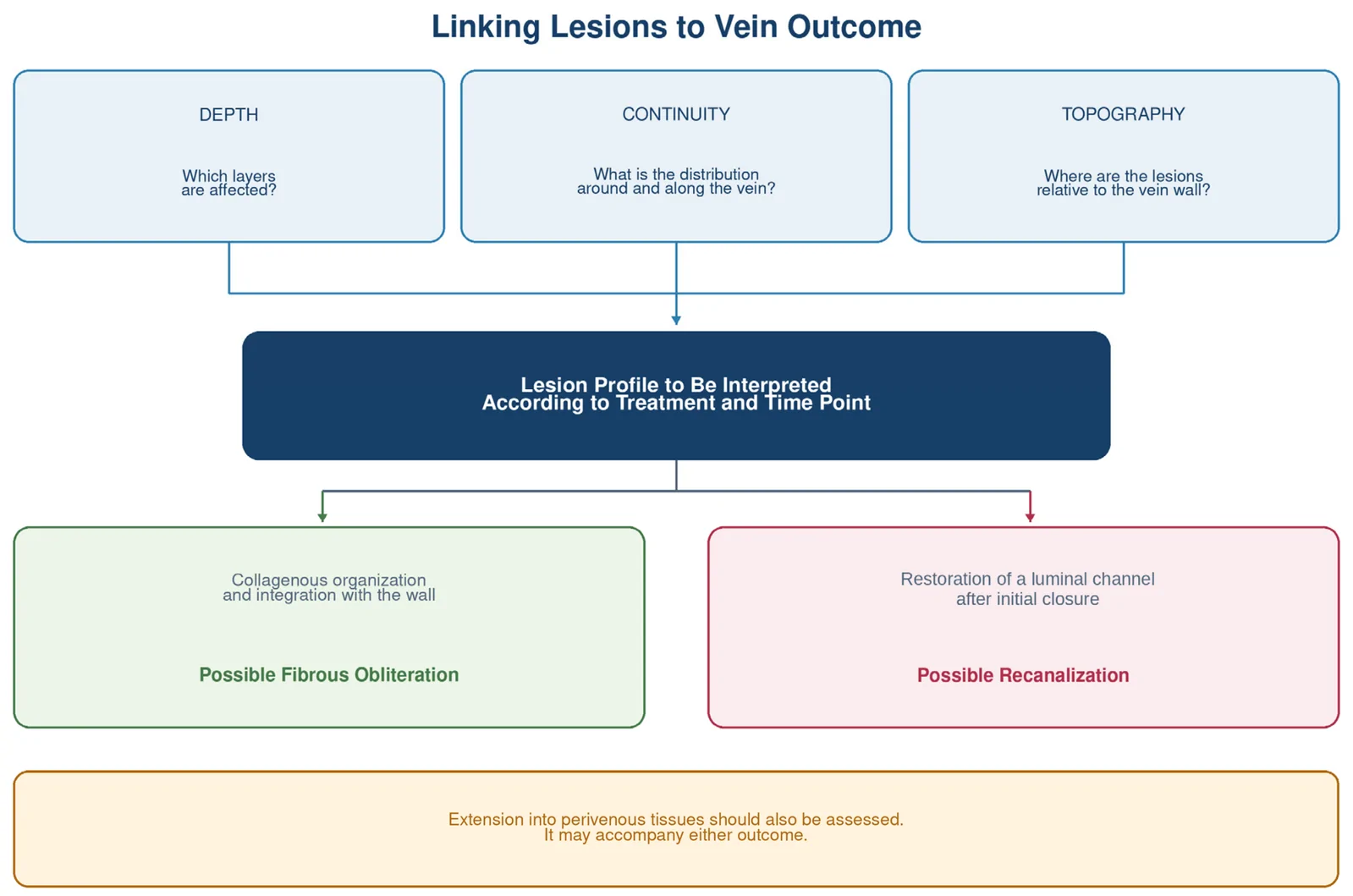
Depth, continuity, and topography: a hypothesis linking lesions to the outcome of the treated vein. The proposed relationships with obliteration and recanalization require validation. Duplex ultrasound follow-up distinguishes reopening after initial closure from persistent patency. Extension into perivenous tissues may accompany either outcome and should be assessed separately; adventitial injury alone does not demonstrate such extension. After cyanoacrylate treatment, biomaterial evolution complements this interpretation.

**Table 1:** Mechanistic Comparison of the Main Modalities.

Modality	Energy/Agent	Initial Event	Primary Target	Inflammatory Signature	Reported or Anticipated Outcome	Main Uncertainty
EVLA	Laser/photothermal injury	Heating, steam, coagulation	Endothelium + media	Inflammation and organization	Organization, fibrosis, diameter reduction; possible recanalization	Actual dose and spatial continuity
RFA	Controlled thermal energy	Collagen denaturation and contraction	Media + endothelium	Post-thermal inflammation	Fibrous organization; possible recanalization	Contact, cycles, geometry
Sclerotherapy	Sclerosant, particularly a detergent	Membrane toxicity	Endothelium, followed by variable medial injury	Inflammatory/apoptotic response varying by agent	Fibrosis or recanalization	Agent–wall contact and blood dilution
MOCA	Mechanical action + detergent	Abrasion, spasm, injection	Endothelium + medial penetration	Amplified chemical response	Reported fibrosis; possible endothelial restoration and recanalization	Relative contribution of the two components
Cyanoacrylate	Polymerizing adhesive	Apposition and polymerization	Lumen/material–wall interface	Macrophages, giant cells, granuloma	Encapsulation and fibrosis; variable material persistence	Formulation, time point, and sampling context
HIFU	Focused ultrasound: thermal effect or cavitation	Focal coagulation or mechanical injury	Wall at the acoustic focus	Dose-dependent inflammation	Thermal: experimental remodeling; mechanical: short-term occlusion and fibrosis	Depth, selectivity, perivenous injury, and durability

The trajectories presented are descriptive. They constitute neither a quantitative comparison of efficacy nor a validated prediction of durable closure.

**Table 2:** (**A**) Thermal and Acoustic Studies. (**B**) Chemical, Mechanochemical, and Adhesive Studies.

(A)					
Ref.	Modality/Model	Documented Biological Material	Time Point	Methods	Main Contribution
[3]	EVLA 980 nm/RFA, bovine ex vivo	Bovine foot veins; histology: 4 segments (2/technique)	Immediate	H&E	Homogeneous RFA lesions in this model; focal and sometimes perforating laser lesions.
[4]	EVLA 808 nm, human in vivo	16 patients, 24 limbs, 29 specimens	Immediate	Histology	Frequent intimal injury, with variable depth and continuity.
[5]	EVLA 980/1500 nm, caprine	14 goats, 28 veins	Immediate to 3 weeks	Sequential histology	Organization and recanalization in unevenly injured areas.
[6]	EVLA with/without centering, caprine	20 veins: 10 with centering, 10 with bare fiber	Immediate, day 10, 3 weeks	Histology	Centering and homogeneity of lesion distribution.
[7]	EVLA 1500 nm, caprine	12 goats, 24 veins	Day 7	Semiquantitative histology	Influence of residual blood, position, and tumescence.
[8]	EVLA 1920 nm, human ex vivo	1 GSV tributary divided into 5 sections	Acute assessment	Histology/IF	Relationship between dose and wall injury; single human specimen.
[9]	EVLA 1910 nm, ovine	Veins from Edilbay sheep	Immediate, day 30, 3 months	Histology/duplex ultrasound	Dose-dependent connective tissue transformation with incomplete occlusion.
[27]	EVLA 1470-nm radial fiber/RFA, human ex vivo	Human saphenous segments	Immediate	Azan trichrome/morphometry	Denaturation patterns and depth according to device; no healing follow-up.
[10]	Steam, RFA, laser; caprine	6 goats, 12 veins	Day 7	Comparative histology	Comparison of short-term thermal lesions.
[11]	RFA, canine	8 dogs; iliofemoral veins	Not reported	Histology/coagulation	Intimal injury and inflammation; occlusion or stenosis in this model.
[22]	Thermal HIFU, human in vitro	20 saphenous segments	Immediate	Contraction/histology	Wall retraction; no evidence of durable closure.
[23]	Thermal HIFU, ovine	3 animals per follow-up group	Days 30, 60, and 90	Duplex ultrasound/histology	Occlusion and remodeling; dose and perivenous protection are critical.
[24]	Mechanical HIFU, ovine	Collateral saphenous vein	Day 7	Duplex ultrasound/histology	Feasibility of cavitation-induced occlusion without injected microbubbles.
[25]	Mechanical HIFU, ovine	2 ewes, 4 veins	Day 10	Histology	Intimal injury and medial fibrosis; protocol-dependent external involvement.
[50]	EVA, EVLA, foam; ovine	8 venous axes: 6 iliofemoral, 2 jugular	Acute	H&E, Masson trichrome, morphometry	Residual intima and media; does not predict late closure.
[30]	Laser and 3% polidocanol foam, human	32 patients: laser 9, foam 11, electric welding 12	Not reported	H&E, van Gieson; CD34, α-SMA, vimentin	Wall injury and marker distribution; laser and foam arms extracted, with insufficient sampling-time and laser-parameter detail.
[31]	RFA ClosureFAST/VENISTAR, canine	7 dogs; 23 veins (11/12); 69 analysis sites	Immediate: 1 dog; day 14: 6 dogs	H&E; TTC	Depth and distribution comparison; no tumescence at immediate assessment, tumescence at day 14: time and protocol effects are confounded.
[28]	1470-nm radial laser, human ex vivo	1 donor; GSV; triplicates and 10 fields/slide	Acute; 7 W, pullback 1 mm/s	Histology, elastic-fiber staining, trichrome; α-SMA	Pilot model of immediate injury; triplicates and fields are not independent patients.
[29]	980-nm laser, human ex vivo	11 veins; number of distinct donors not reported; paired halves	Acute	H&E; CCND1, BCL2, BAX	Intimal injury and gene expression; transcripts do not establish functional survival.
[46]	Pulsed HIFU + microbubbles + fibrinogen, rabbit	7 rabbits; 6 assessed at day 14	Acute and day 14	Histology; occlusion follow-up	Acute occlusion of all 7 segments; at day 14: 4 nonocclusive thrombi, 2 without thrombus; combined technique.
(**B**)					
**Ref.**	**Modality/Model**	**Documented Biological Material**	**Time Point**	**Methods**	**Main Contribution**
[12]	Sclerosants, human in vitro	Human saphenous segments	10 min	Histology	Direct effects of solutions; model without in vivo healing.
[13]	3% STS foam, human in vivo	6 patients	2, 15, and 30 min	Histology	Endothelial denudation and early thrombus formation.
[14]	STS foam ± denudation, human	Specimens from 20 patients	Acute	Quantitative histology	Endothelial loss and medial injury according to contact conditions.
[15]	STS/polidocanol, human ex vivo	Varicose vein segments	1/10 min; 24-h culture	IF: CD31, α-actin, p53, ICAM-1	Delayed response after STS; differences from polidocanol.
[26]	Polidocanol foams, human in vitro/ex vivo	Human endothelial cells and umbilical veins	Acute assessment	Cell adhesion/permeability	Functional endothelial effects; no fibrosis follow-up.
[32]	Experimental AEO15-TA/PVA foam	HUVECs; mouse tail veins and rabbit auricular veins	24 h, day 7, or day 12 depending on analysis	H&E/IF/Masson trichrome/protein analyses	Vascular injury and short-term collagen deposition; preclinical data.
[16]	MOCA/ClariVein^®^, caprine	11 goats, 12 veins; 5 MOCA + STS	12 weeks	H&E/Masson trichrome	Fibrous obliteration of veins treated with MOCA + STS.
[17]	MOCA/sclerosant, human ex vivo	Human extrafascial GSVs	10–11 min	H&E/IF	Greater medial injury with combined mechanical treatment and STS.
[18]	MOCA, human ex vivo	24 patients; 24 GSV specimens	Acute	H&E, Masson trichrome, IHC	Comparison of saline, polidocanol, mechanical treatment alone, and MOCA.
[38]	MOCA/ClariVein^®^, human	1 patient, 1 GSV	1 year	Histology/IHC	Fibrous obliteration with endothelial loss and medial injury; isolated case.
[39]	MOCA/Flebogrif^®^ + 3% polidocanol, ovine	9 sheep in 3 groups; lateral saphenous veins	1 month	H&E/Masson–Goldner/histometry	Restored endothelium, new wall collagen, and little adventitial change; functional outcome not demonstrated.
[19]	Histoacryl^®^/Lipiodol^®^, human	6 patients	6 days to 3 years	Histopathology	Foreign-body reaction; material not identifiable at 3 years in this series.
[20]	VenaSeal™, human	1 patient, 1 vein	5.5 years	Histology	Fibrosis, polymer remnants, giant cells, and granulomas.
[21]	VenaSeal™, human	1 patient, 1 vein	2 years	Histology	Largely preserved media; giant cells without adventitial infiltration.
[40]	Cyanoacrylate, human; persistent hypersensitivity	1 female patient, 1 excised GSV	Day 200	Histology/lymphocyte IHC	Predominantly luminal, CD4-predominant infiltrate; complication setting.
[41]	Cyanoacrylate/VenaSeal, human	1 patient; GSV and surrounding tissues	Day 77	H&E	Residual glue, giant cells, and scar collagen; complication specimen, without frequency estimation.
[42]	Cyanoacrylate, human	2 patients with puncture-site histology; clinical cohort of 511 patients	7 months in the illustrated case; second time point not reported	Histology	Puncture-site granulomas; the 511 patients are not the histological sample size.
[43]	Cyanoacrylate, human	1 patient; excised GSV	Complication at 3 months; exact sampling time not reported	H&E	Endothelial destruction, neutrophils, granuloma, and fistula; aseptic complication with negative cultures.
[30]	Laser and 3% polidocanol foam, human	32 patients: laser 9, foam 11, electric welding 12	Not reported	H&E, van Gieson; CD34, α-SMA, vimentin	Wall injury and marker distribution; laser and foam arms extracted, with insufficient sampling-time and laser-parameter detail.
[44]	Cyanoacrylate, human	1 original histological case among 2 author-reported clinical cases	Symptoms at about 3 months; exact histology time not reported	Histology	Inflammation and foreign-body reaction in venous and perivenous tissues; only original case data are extracted from this review.
[33]	2% polidocanol foam ± 72% glycerine, human ex vivo	6 patients; 3 paired segments per GSV	5-min contact	H&E; CD31, α-SMA	Acute endothelial and medial effects; paired segments are not additional patients.
[45]	Cyanoacrylate, human	1 female patient; subcutaneous mass separate from the GSV	9 months	Histology	Glue, giant cells, plasma cells, and lymphocytes; extravascular specimen rather than saphenous-wall histology.
[34]	Ethanol, ethanolamine oleate, 1% polidocanol, rat	61 rats entered; 7 excluded after death; sample sizes vary by analysis	24 h and day 7; electron microscopy at day 7	H&E, EVG; electron microscopy	Low-flow femoral-vein model; venous injury, patency, and nerve effects assessed separately.
[35]	Liposomal bleomycin/1% STS/saline, rabbit	18 rabbits; 3 groups of 6	1 h to day 45; day 1/day 2 discrepancy between text sections	H&E	Inflammation, vascular injury, thrombosis, and fibrosis; venous findings distinguished from arteriolar images.
[36]	1%, 5%, 20%, 50% phenol/1% STS/saline, rabbit	35 animals reported; 6 groups of 5 described: unresolved inconsistency	1 h to day 45; day 1/day 2 discrepancy between text sections	H&E	Wall injury, organized thrombus, and fibrosis; experimental agent, without extrapolation to human efficacy or safety.
[37]	Triplex: sodium salicylate, lidocaine, glycerine ± foam, human	Same patient and segment illustrated; total histological sample size not reported	Before and 30 days after treatment	Histological illustration; stain not reported	Remodeling without occlusion; the 4000 clinical patients are not a histological sample size; low-level illustrative evidence.

The numbers of subjects, veins, and segments are not interchangeable. Sample sizes are reported when they could be verified; no value was inferred from the number of sections or images. GSV: great saphenous vein; H&E: hematoxylin and eosin; IF: immunofluorescence; IHC: immunohistochemistry; EVA: empty vein ablation; STS: sodium tetradecyl sulfate; PVA: polyvinyl alcohol; TA: tranexamic acid; AEO15-TA: surfactant–tranexamic acid conjugate; HUVEC: human umbilical vein endothelial cells.

**Table 3:** Proposed Core Assessment and Additional Investigations.

Domain	Primary Measure	Proposed Technique/Marker	Recommended Unit	Interpretation and Limitation
Lumen	Area, occlusion, channels	H&E/digital segmentation	mm^2^ and %	Distinguish organized tissue, biomaterial, and luminal channel
Endothelium	Continuity/restoration	vWF or CD31/ERG; morphology	% of circumference	Distinguish luminal lining from reparative microvessels
Intima	Thickness and subintima	H&E/morphometry	µm, median and maximum	Quantify hyperplasia and organization
Media	Architecture, thickness, phenotype	H&E, Masson trichrome, α-SMA	µm and % of wall affected	α-SMA alone does not demonstrate cell viability
Adventitia	Inflammation and fibrosis	H&E, Masson trichrome	score + area	Assess reparative response and transmural extension
Immunity	Macrophages/giant cells	CD68 ± CD163; morphology	cells/mm^2^	Interpret according to time point and compartment; polarization cannot be inferred from a single marker
Matrix	Matrix composition/organization	Masson trichrome; Sirius red/EVG; MMP–TIMP as adjuncts	% area/intensity	MMP expression ≠ enzymatic activity; thresholds not validated
Adjacent tissues	Perivenous injury	H&E/anatomical score	distance and grade	Document selectivity and safety
Imaging	Diameter, flow, compressibility	Longitudinal duplex ultrasound; end-point histology	mm and prespecified categories	Relate the identified segment to its tissue status at sampling

Proposed core panel: H&E, morphometry, collagen staining, an endothelial marker, and α-SMA. Additional analyses should be tailored to the scientific question and species. This panel is neither a score nor a validated recommendation. EVG: elastin van Gieson stain; MMP: matrix metalloproteinase; TIMP: tissue inhibitor of metalloproteinases.

## Data Availability

No new data were created or analyzed in this study. Data sharing is not applicable to this article.

## Supplementary Materials

The following supporting information can be downloaded at https://www.mdpi.com/article/10.3390/medsci14060691/s1. Supplementary Material S1: PubMed strategy, description of study selection, and flow diagram. The list of abbreviations is provided at the end of the document.

## Abbreviations

**Abbreviation**	**Definition**	**Abbreviation**	**Definition**
**AEO15-TA**	Tranexamic acid–fatty alcohol polyoxyethylene ether conjugate	**IF**	Immunofluorescence
**CD4**	Cluster of differentiation 4; T-lymphocyte marker	**IHC**	Immunohistochemistry
**CD31**	Cluster of differentiation 31; endothelial marker	**IMVIA**	Imaging and Artificial Vision
**CD68**	Cluster of differentiation 68; macrophage marker	**MeSH**	Medical Subject Headings; controlled biomedical vocabulary of the National Library of Medicine
**CD163**	Cluster of differentiation 163; macrophage marker	**MMP**	Matrix metalloproteinase
**CHU**	French university hospital (centre hospitalier universitaire)	**MOCA**	Mechanochemical ablation
**ERG**	ETS-related gene transcription factor; endothelial nuclear marker	**PMID**	PubMed identifier; unique identifier for a PubMed record
**EVA**	Empty vein ablation	**PVA**	Polyvinyl alcohol
**EVG**	Elastin van Gieson stain	**RFA**	Radiofrequency ablation
**EVLA**	Endovenous laser ablation	**STS**	Sodium tetradecyl sulfate
**GSV**	Great saphenous vein	**TA**	Tranexamic acid
**H&E**	Hematoxylin and eosin	**TIMP**	Tissue inhibitor of metalloproteinases
**HIFU**	High-intensity focused ultrasound	**vWF**	von Willebrand factor
**HUVEC**	Human umbilical vein endothelial cell	**α-SMA**	Alpha-smooth muscle actin
**ICAM-1**	Intercellular adhesion molecule 1		
