# Supplementary material for: Understanding Venous Remodeling After Minimally Invasive and Noninvasive Treatments for Varicose Veins: A Mechanistic Narrative Review
Source: Med Sci (Basel). 2026 Oct 21;14(6):691. doi: 10.3390/medsci14060691 (PMC13706723; doi:10.3390/medsci14060691)
Supplement: Supplementary file 1 [file medsci-14-00691-s001.zip › medsci-4598491-supplementary.pdf]

medical sciences

MDPI

Review

Understanding Venous Remodeling After Minimally Invasive and Noninvasive Treatments for Varicose Veins: A Mechanistic Narrative Review

SUPPLEMENT FILE

Supplementary Material S1. Documented PubMed Search

The search and selection strategy resulted in the inclusion of 45 distinct tissue-based publications in this synthesis; one publication is represented in both parts of Table 2 and counted only once.

Database searched: PubMed, without restriction to MEDLINE-indexed references.

Search date: 6 September 2026. Publication period: through 31 August 2026, with no restrictive lower date limit.

Filters: No restrictions on language, species, or study type. The limit concerned the publication date; the search was run on 6 September 2026.

Result: 698 publications identified, corresponding to 698 unique PubMed identifiers (PMIDs).

Main Keywords

Veins: “varicose veins”, “venous insufficiency”, “saphenous vein”. Treatments: “laser”, “radiofrequency”, “sclerotherapy”, “mechanochemical ablation”, “cyanoacrylate”, “focused ultrasound”. Tissue responses: “histology”, “immunohistochemistry”, “fibrosis”, “endothelium”.

Complete Search String for Reproducibility

("Varicose Veins"[MeSH Terms] OR "Venous Insufficiency"[MeSH Terms] OR "Saphenous Vein"[MeSH Terms] OR saphen*[Title/Abstract] OR varicos*[Title/Abstract] OR endovenous[Title/Abstract] OR "superficial vein*"[Title/Abstract] OR "superficial venous"[Title/Abstract] OR "venous reflux"[Title/Abstract] OR "vein wall"[Title/Abstract] OR "venous wall"[Title/Abstract]) AND ("Laser Therapy"[MeSH Terms] OR "Catheter Ablation"[MeSH Terms] OR "Sclerotherapy"[MeSH Terms] OR laser*[Title/Abstract] OR radiofrequen*[Title/Abstract] OR sclerotherap*[Title/Abstract] OR sclerosant*[Title/Abstract] OR polidocanol[Title/Abstract] OR "sodium tetradecyl sulfate"[Title/Abstract] OR "sodium tetradecyl sulphate"[Title/Abstract] OR mechanochem*[Title/Abstract] OR ClariVein[Title/Abstract] OR cyanoacrylat*[Title/Abstract] OR VenaSeal[Title/Abstract] OR Histoacryl[Title/Abstract] OR "focused ultrasound"[Title/Abstract] OR "high intensity ultrasound"[Title/Abstract] OR HIFU[Title/Abstract] OR histotrips*[Title/Abstract] OR steam[Title/Abstract] OR ablat*[Title/Abstract]) AND ("Histological Techniques"[MeSH Terms] OR "Immunohistochemistry"[MeSH Terms] OR "Microscopy, Electron"[MeSH Terms] OR histolog*[Title/Abstract] OR histopatholog*[Title/Abstract] OR immunohistochem*[Title/Abstract] OR immunofluorescen*[Title/Abstract] OR morpholog*[Title/Abstract] OR morphometr*[Title/Abstract] OR ultrastructur*[Title/Abstract] OR microscop*[Title/Abstract] OR "vein wall"[Title/Abstract] OR "venous wall"[Title/Abstract] OR endotheli*[Title/Abstract] OR collagen*[Title/Abstract] OR fibros*[Title/Abstract] OR fibrotic[Title/Abstract] OR "foreign body"[Title/Abstract] OR

macrophag*[Title/Abstract] OR "matrix metalloproteinase"[Title/Abstract]) AND ("0001/01/01"[Date - Publication] : "2026/08/31"[Date - Publication])

Selection Status

After the initial screening of the 698 identified references, 537 were excluded on the basis of title and abstract, 25 were directly referred for full-text assessment, and 136 required additional verification.

Of these 136 references, 107 were excluded after detailed reassessment of the title and abstract, mainly because they lacked directly usable histopathological or immunohistochemical data, used a model or technique outside the review scope, or made an insufficiently direct contribution to the question of venous remodeling. The remaining 29 references underwent full-text assessment.

A total of 54 full-text articles were therefore assessed: 25 publications directly referred for assessment and 29 publications retained after additional verification. Following full-text assessment, 45 distinct tissue-based publications were included and nine were not included. The nine individual reasons for non-inclusion are not itemized in this supplement. Table 2A contains 20 publication entries and Table 2B contains 26; one publication covering both laser and foam is included in both tables and counted once in the total of 45.

Selection stage / synthesis content		Number
PubMed publications identified (unique PMIDs)		698
References excluded after title and abstract assessment		644
Full-text articles assessed		54
Publications not included after full-text assessment	9	
Tissue-based publications included in the synthesis	45	
Clinical-context or guideline references	6	
Total bibliographic references in the synthesis	51	

Literature Search and Study Selection

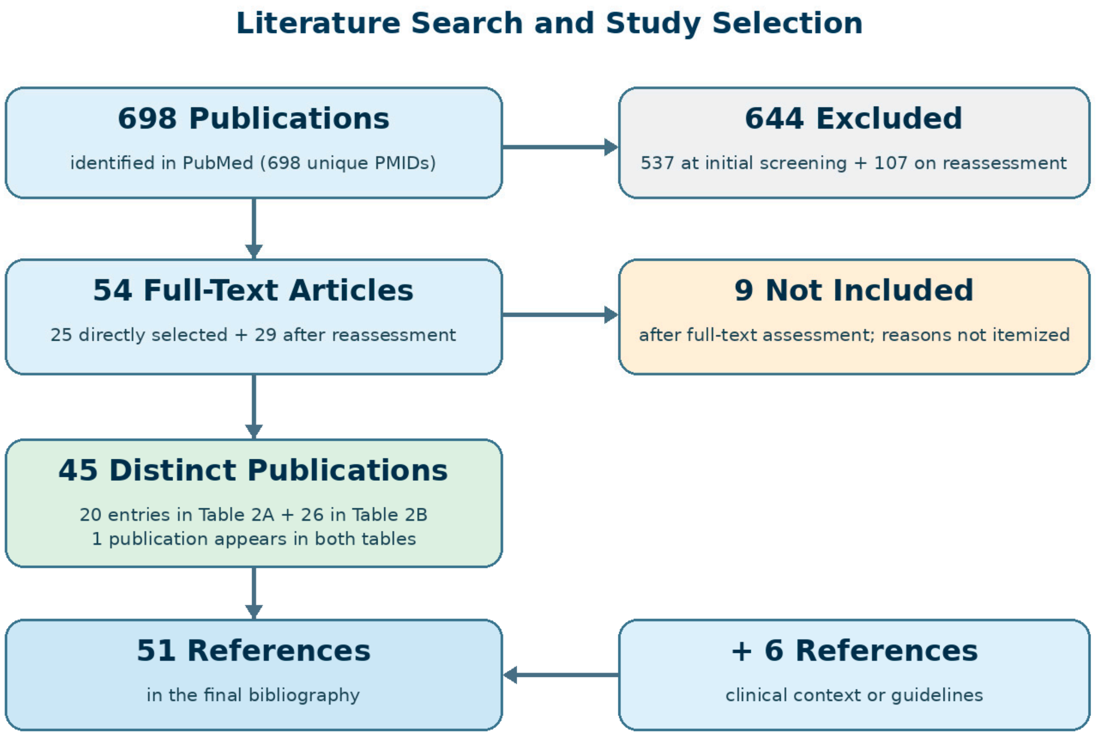
Literature Search and Study Selection
698 Publications
identified in PubMed (698 unique PMIDs)
644 Excluded
537 at initial screening + 107 on reassessment
54 Full-Text Articles
25 directly selected + 29 after reassessment
9 Not Included
after full-text assessment; reasons not itemized
45 Distinct Publications
20 entries in Table 2A + 26 in Table 2B
1 publication appears in both tables
51 References
in the final bibliography
+ 6 References
clinical context or guidelines

This synthesis therefore includes 45 distinct tissue-based publications and six clinical-context or guideline references, for a total of 51 bibliographic references. The strategy documents a mechanistic narrative review based on a structured literature search rather than an exhaustive systematic review.
